# Risk of mortality after emergency department presentation for self-harm among children and adolescents in Wales: a population-based cohort study

**DOI:** 10.1016/j.eclinm.2026.104230

**Published:** 2026-09-28

**Authors:** Gabrielle M. Beaudry, Kirstie O'Hare, Rongqin Yu, Seena Fazel, Ian Kelleher

**Affiliations:** a Faculty of Medicine and Health Sciences, McGill University, Montréal, Québec, Canada; b Centre for Outcomes Research and Evaluation, Research Institute of the McGill University Health Centre, McGill University Health Centre, Montréal, Québec, Canada; c Institute for Neuroscience and Cardiovascular Research, University of Edinburgh, Edinburgh, UK; d Discipline of Psychiatry and Mental Health, University of New South Wales, Sydney, Australia; e Department of Psychiatry, University of Oxford, Warneford Hospital, Oxford, UK; f Oxford Health NHS Foundation Trust, Warneford Hospital, Oxford, UK; g School of Medicine, University College Dublin, Dublin, Ireland; h Faculty of Medicine, University of Oulu, Oulu, Finland; i St John of God Hospitaller Service Group, Stillorgan, Dublin, Ireland

**Keywords:** Self-harm, Adolescents, Emergency department, Mortality, Suicide, Population-based cohort

## Abstract

**Background:**

Self-harm in childhood and adolescence is associated with substantial morbidity and premature mortality, particularly from suicide. We aimed to quantify the risk of cause-specific and all-cause mortality following emergency department (ED) presentations for self-harm.

**Methods:**

In this retrospective population-based cohort study, we used anonymised, linked electronic health records from the Secure Anonymised Information Linkage (SAIL) Databank. We included children and adolescents aged 5–18 years who presented to EDs in Wales between 2008 and 2023. Individuals with at least one ED presentation for self-harm were assigned to the exposed group, and those with no recorded self-harm presentations formed the comparison group. Follow-up began at the index ED presentation and continued until death, possible emigration, or end of data availability. The primary outcome was suicide; secondary outcomes included accidental death, other causes of death, and all-cause mortality. Associations between self-harm and cause-specific mortality were estimated using Cox proportional hazards models adjusted for age and sex. Kaplan–Meier failure curves (1 − survival) were estimated separately for each cause of death.

**Findings:**

Between 2008 and 2023, we identified 611,336 young people who presented to EDs, of whom 14,007 (2.3%) had at least one self-harm presentation. During follow-up, 1451 deaths were recorded. Mortality was higher among those presenting with self-harm compared with controls without self-harm presentations (114 [0.8%] vs 1337 [0.2%], adjusted hazards ratio [aHR] 3.1, 95% CI 2.6–3.8). Suicide risk was markedly elevated in the first year after presentation (aHR 13.4, 95% CI 4.7–38.0) and remained increased during follow-up (aHR 4.9, 95% CI 3.4–6.9). There was a similarly increased risk of accidental death within one year (aHR 13.9, 95% CI 6.2–31.1) and after one year (aHR 5.5, 95% CI 3.9–7.7). No clear association between self-harm and other causes of death was observed.

**Interpretation:**

ED presentation for self-harm identifies young people at elevated risk of premature mortality. Preventive interventions should extend beyond suicide alone to address accidental deaths, particularly in the period immediately following presentation.

**Funding:**

McGill Global Health Programs Fellowship; John Fell Fund; 10.13039/501100000272NIHROxford Health Biomedical Research Centre; 10.13039/501100000691Academy of Medical Sciences; UK Department for Business, Energy and Industrial Strategy.

**Research in context**

**Evidence before this study**

We searched PubMed from inception to 4 February 2026, using combinations of MeSH terms and free-text keywords related to self-harm, children and adolescents, emergency department (ED) presentation, and mortality, restricting results to emergency care settings. We identified several relevant cohort studies. One Canadian cohort study of adolescents presenting with self-poisoning reported substantially increased long-term risks of suicide, accidental death, and all-cause mortality. Another Canadian investigation showed that adolescents presenting to EDs with self-harm had higher risks of subsequent mortality, suicide, and repeat self-harm, alongside greater health-care use and costs, than matched controls. A multicentre English prospective study of more than 9000 young people found persistently elevated risks of suicide and accidental death following self-harm. A population-based registry study from Northern Ireland similarly reported a near 20-fold increased risk of suicide after ED presentation for self-harm. However, these studies focused primarily on suicide or specific methods of self-harm, were restricted to selected centres or regions, or did not comprehensively examine cause-specific mortality across the full child and adolescent age range of 5–18 years.

**Added value of this study**

To our knowledge, this is one of the largest population-based cohorts of children and adolescents presenting to EDs with self-harm to examine subsequent cause-specific mortality. Using linked national routine data and long-term follow-up, we simultaneously examined suicide, accidental mortality, other causes of death, and all-cause mortality across the full child and adolescent age range, including younger children. These findings extend previous studies by providing nationally representative estimates across a broader developmental range and multiple causes of death within a single cohort.

**Implications of all the available evidence**

ED presentation for self-harm identifies young people at high risk of premature death from both suicide and accidental causes. Prevention strategies should extend beyond suicide alone and prioritise early, developmentally-informed and consistent follow-up care. Risk patterns may differ in younger children, but evidence in this age group remains scarce and warrants targeted research.

## Introduction

Self-harm, defined as intentional self-injury or self-poisoning irrespective of suicidal intent,^1^ represents a major public health concern among children and adolescents and is a key risk factor for subsequent suicide in this population.^2^^,^^3^ Approximately one in six adolescents reports a lifetime history of self-harm in community-based studies, highlighting its substantial public health burden.^4^ Despite its clinical and public health significance, self-harm has historically received less attention than suicide within international and governmental prevention policies.^5^ Recurrent self-harm is common among young people,^6^ and children and adolescents presenting to clinical services following self-harm experience a substantially elevated risk of subsequent suicide compared with the general population.^2^^,^^3^

Emerging evidence indicates that self-harm in young people is associated with an increased risk of premature mortality extending beyond suicide alone. Population-based studies from England, Northern Ireland, and Canada have reported elevated risks of death from accidental causes following self-harm.^2^^,^^7^, ^8^, ^9^, ^10^ However, few studies have investigated specific causes of mortality within the same cohort, particularly in younger children. Much of the existing literature has examined suicide and non-suicide mortality separately, despite growing evidence that both contribute to excess mortality following self-harm.^6^, ^7^, ^8^ Examining these outcomes within the same cohort is important, as differences in classification and reporting practices across settings might influence whether deaths are recorded as suicide or accidents, while prevention strategies differ by cause. Recent meta-analytic evidence of a high prevalence of suicidal ideation and self-harm behaviours among preadolescents highlights the need for research spanning the full developmental range from childhood through late adolescence.^11^^,^^12^

Adolescence, particularly later adolescence, is characterised by a sharp increase in self-harm.^2^^,^^13^ Young women are disproportionately more likely to engage in self-harm, a sex difference that has persisted over the past four decades.^13^, ^14^, ^15^ However, among those who self-harm, the risk of subsequent suicide is nearly two-fold higher in males than in females,^8^^,^^10^ and is greater among older adolescents (aged 16–18 years) compared with younger (aged 10–15 years). Sex differences may be even more pronounced for accidental mortality, with one UK study reporting rates of accidental death approximately four times higher in males than females following self-harm.^2^

In Wales, suicide was the leading cause of death among individuals aged 5–19 years in 2024, accounting for 14.9% of all deaths, followed by homicide (8.1%) and land transport incidents (6.8%).^16^ Among young people, self-harm frequently leads to contact with acute health-care services, most commonly through emergency departments (EDs). These presentations therefore represent an important point in the clinical pathway at which individuals at elevated risk of subsequent mortality can be identified. Improved understanding of the association between self-harm and subsequent suicide, accidental death, and other causes of mortality—including natural and non-accidental external causes—is needed to inform risk stratification, guide targeted interventions, and strengthen evidence-based prevention strategies.

Thus, our aim was to quantify the absolute and relative risks of suicide, accidental death, and all-cause mortality among children and adolescents aged 5–18 years between 2008 and 2023, comparing those with and without self-harm. We further sought to characterise the timing of death after self-harm, and to identify demographic and clinical factors associated with elevated mortality risk.

## Methods

### Study design and participants

We conducted a population-based observational cohort study using anonymised, routinely collected data held within the Secure Anonymised Information Linkage (SAIL) Databank.^17^, ^18^, ^19^, ^20^, ^21^ The SAIL Databank hosts linked datasets from health, social care, and administrative sources for the population of Wales. Wales is one of the four nations of the UK. ED services are broadly similar across the four UK nations, as they are publicly funded and free at the point of access. All datasets are complete, except for the Welsh general practice (GP) data, which covers ∼80% of GP practices in Wales and ∼83% of the overall population.

The source population comprised all individuals aged 5–18 years who presented to any NHS Wales ED between April 16, 2008, and November 1, 2023, the earliest and latest dates available in the dataset, respectively. This age range is consistent with previous population-based studies of ED presentations for self-harm in young people.^2^ From this population, the analytic cohort comprised individuals who were registered with a Welsh GP for at least 12 months before their index ED presentation (indicating that they were ordinarily resident in Wales) and had complete demographic information (age and sex). Records with missing linkage identifiers were excluded. Although all eligible ED records during this period were included, national coverage was incomplete in the earlier years, with partial data available from 2008 and full coverage achieved from 2012 onwards.^22^

Eligible individuals were classified into one of two mutually exclusive exposure groups based on whether they had at least one ED presentation for self-harm during the study period. Follow-up began on the index date and continued until the earliest of death, end of GP registration (used as a proxy for possible emigration), or the end of data availability. Individuals who died on the same day as their index ED presentation were included in descriptive analyses but excluded from survival analyses, as these deaths occurred before follow-up began and therefore did not contribute person-time at risk.

### Exposure

Self-harm presentations were identified using diagnostic and attendance information from the ED dataset (EDDS), which applies a standardised coding framework across EDs in Wales. Although EDDS does not capture the diagnostic granularity of ICD-10 or Read codes, it has successfully been used in previous registry studies to identify self-harm presentations in routine emergency care data. We applied the same case-definition approach used in prior Welsh registry studies.^22^ A presentation was classified as self-harm if the attendance group was recorded as ‘Deliberate Self-Harm.‘^23^ Presentations coded as ‘Undetermined Intent’ were additionally classified as self-harm if at least one accompanying diagnosis met the predefined case-definition for self-injury or poisoning, as described below.^22^

Each ED presentation may include up to six diagnosis types and corresponding anatomical sites; all recorded diagnostic fields were considered jointly when determining self-harm status. Consistent with the published Welsh case definition, diagnoses indicative of self-harm included lacerations involving the forearm or wrist and poisoning or overdose involving prescribed, non-prescribed, illicit, or unspecified substances.^22^ Presentations in which the sole diagnosis was alcohol overdose were excluded unless accompanied by another qualifying self-harm diagnosis, because isolated alcohol overdose is more appropriately classified as acute alcohol intoxication than intentional self-poisoning.^14^ All remaining presentations were classified as non–self-harm related.

### Cohort assignment

Individuals were assigned to the self-harm group if they had at least one recorded ED presentation for self-harm during the study period, whereas those with no recorded self-harm presentations were assigned to the non–self-harm comparison group. The index date was defined as the date of the first self-harm presentation for exposed individuals and the date of the first ED presentation for any other reason in the comparison group. For individuals with multiple ED presentations, exposure classification followed a hierarchical first-occurrence approach that prioritised self-harm; thus, individuals whose initial presentation was for another reason but who subsequently presented with self-harm were classified in the self-harm group, with follow-up anchored at the date of their first self-harm presentation.

### Outcomes

The primary outcome was death by suicide, identified through linkage to the Annual District Death Extract (ADDE). Causes of death were classified according to the International Classification of Diseases, 10th revision (ICD-10). Suicide was defined as death with an underlying cause coded as intentional self-harm (ICD-10 X60–X84) or events of undetermined intent (ICD-10 Y10–Y34), consistent with established suicide epidemiological research and current UK policy practice.^2^^,^^16^^,^^24^

Secondary outcomes included death due to accidental causes (ICD-10 V01–X59), death due to other causes (all remaining ICD-10 codes, including R99 [ill-defined or unspecified causes]), and an overall outcome of all-cause mortality. Where multiple causes of death were recorded, classification was based on the underlying cause.

### Covariates

Age and sex were included as core adjustment variables in all primary comparative analyses. Age was defined at the index ED presentation and modelled as a continuous variable; for descriptive purposes, age was also categorised as 5–9, 10–14, and 15–18 years, broadly corresponding to stages of childhood and adolescence. Age was treated as a fixed baseline characteristic, with individuals remaining under follow-up beyond age 18 until death, possible emigration, or the end of data availability. Sex was coded as male or female at the index ED presentation.

Within the self-harm cohort, additional demographic, social, and clinical characteristics were examined as potential risk factors for subsequent mortality. These included area-level deprivation; Looked After Child status, defined as being in the care of local authorities and identified using the Looked After Children Wales dataset; recent and lifetime mental health service contact, defined as any inpatient or outpatient contact with Child and Adolescent Mental Health Services (CAMHS), assessed within the 12 months preceding the index presentation and across the individual's recorded clinical history^25^; and urban–rural classification derived from Lower Super Output Area 2011 codes. Ethnicity was not included as a covariate because ethnicity data were unavailable for a large proportion of participants, consistent with known limitations in ethnicity recording in UK electronic health records during this period.^26^

Clinical characteristics included recurrent self-harm, defined as one or more additional ED presentations for self-harm occurring after the index event and before the end of follow-up, coded as a binary variable. Hospital admission within seven days of the index self-harm presentation, identified through linkage to inpatient records, was used as a proxy indicator of clinical severity; deaths occurring during this seven-day period were retained in the analyses.

### Statistical analysis

Baseline characteristics were summarised separately for the self-harm and control groups (individuals with no recorded ED presentation for self-harm). Continuous variables were reported as medians with interquartile ranges, and categorical variables as counts and percentages. Event counts, total follow-up time, and mortality rates per 100,000 person-years were calculated for each exposure group. Categories with fewer than ten events were suppressed or aggregated, in accordance with SAIL disclosure control policies.

Time-to-event analyses were used to examine the association between self-harm presentation and subsequent mortality. Follow-up began on the index date and continued until death, emigration, or the end of data availability, whichever occurred first. Cox proportional hazards models were used to estimate adjusted hazard ratios (aHRs) and 95% confidence intervals (CIs) for primary and secondary outcomes. Models included exposure group (self-harm vs control) and were adjusted for age and sex. Proportional hazards assumptions were assessed using Schoenfeld residuals^27^—where violations were identified, time-dependent effects and/or stratification were applied as appropriate.

For descriptive purposes, cumulative event curves were estimated as one minus the Kaplan–Meier survival estimate for each cause of death separately.

Among individuals presenting with self-harm, additional analyses examined demographic, social, and clinical factors associated with mortality. Poisson regression models were used to estimate incidence rate ratios (IRR) and 95% CIs for primary and secondary outcomes, accounting for variation in follow-up time. This approach is suitable for rare event outcomes with varying follow-up durations and directly estimates rate ratios. All prespecified risk factors were included simultaneously to estimate their independent associations.

Several sensitivity analyses were conducted to assess the robustness of findings. These included modelling recurrent self-harm as a time-varying exposure, whereby individuals contributed person-time to the non-recurrent state until their first recurrent self-harm presentation and to the recurrent state thereafter; reclassifying deaths of undetermined intent (ICD-10 Y10–Y34) as non-suicide deaths^28^^,^^29^; and repeating analyses using complete-case data only.

The study is reported in accordance with the Strengthening the Reporting of Observational Studies in Epidemiology (STROBE) guidelines.^30^ Data were extracted from the SAIL Databank using Structured Query Language (SQL; DB2 version 11.1.4.7 for Windows). All analyses were conducted in R (version 4.5.1) and RStudio.

### Ethics

In accordance with the Health Research Authority guidance, ethical approval is not required for studies that use only anonymised data. Approval to access the SAIL databank was granted by the SAIL Information Governance Review Panel (IGRP #1635) on 01/11/2023. As this research uses deidentified data, individual consent was not required.

### Role of the funding source

The funder had no role in study design, data collection, data analysis, data interpretation, or writing of the manuscript.

## Results

Between 16 April 2008 and 1 November 2023, 2,308,005 ED presentations were recorded in Wales among 669,804 individuals aged 5–18 years. After excluding records without valid person-level linkage or with incomplete diagnostic, demographic, or area-level information, the analytic sample comprised 611,336 individuals (14,007 with at least one ED presentation for self-harm and 597,329 without), contributing 2,101,726 ED presentations overall (Fig. 1). Median cohort follow-up was 9.2 years (IQR 4.9–12.3). An additional 131 individuals who died on the same day as their index ED presentation were included in descriptive analyses of baseline characteristics. Within the self-harm group, the number of ED presentations for self-harm per individual ranged from one to 66, with 10,578 individuals (75.5%) having a single recorded presentation during the study period.

**Fig. 1:**
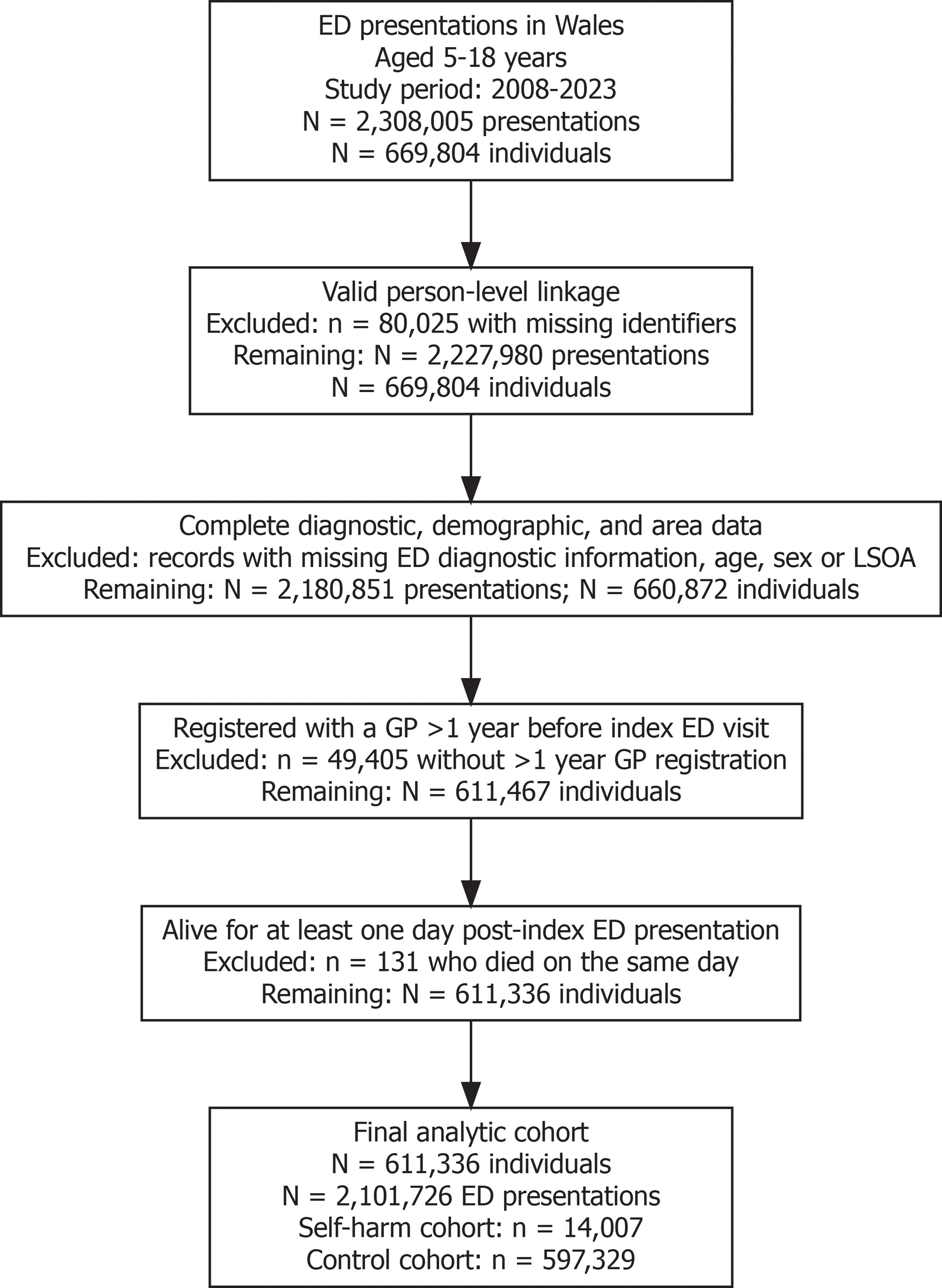
**Study flowchart.** Note. Abbreviations: ED, Emergency Department; GP, General Practitioner; LSOA, Lower Layer Super Output Area.

Baseline characteristics are summarised in Table 1. Individuals in the self-harm group were older at index ED presentation, with more than half aged 15–18 years, whereas the comparison group was predominantly composed of children aged 5–9 years. Females accounted for a greater proportion of the self-harm group than the comparison group. Individuals presenting with self-harm were more likely to reside in more deprived areas and to live in urban settings. Indicators of social and clinical vulnerability were substantially more common in the self-harm group, including Looked-After Child status and contact with CAMHS, both in the year preceding the index presentation and over the lifetime. Hospital admission within seven days of presentation occurred more frequently following self-harm than after other ED presentations. Patterns of recurrent ED use were observed in both groups, but were modestly more frequent among individuals in the self-harm group.

**Table 1:** Baseline characteristics.

Characteristics	Self-harm (N = 14,012)	Control (N = 597,455)
Age group, years		
5–9	480 (3.4%)	331,128 (55.4%)
10–14	6170 (44.0%)	169,719 (28.4%)
15–18	7362 (52.5%)	96,608 (16.2%)
Sex (female)	9299 (66.4%)	274,141 (45.9%)
Deprivation quintile		
1 (most deprived)	4279 (30.5%)	147,693 (24.7%)
2	3443 (24.6%)	123,965 (20.7%)
3	2580 (18.4%)	116,775 (19.5%)
4	1926 (13.7%)	100,302 (16.8%)
5 (least deprived)	1718 (12.3%)	105,762 (17.7%)
Missing	66 (0.5%)	2958 (0.5%)
Urban residence	10,415 (74.3%)	419,954 (70.3%)
Looked-After children status	1980 (14.1%)	13,555 (2.3%)
CAMHS contact		
Previous year	4516 (32.2%)	15,413 (2.6%)
Lifetime	9734 (69.5%)	98,429 (16.5%)
Hospital admission (within 7 days)	6264 (44.7%)	63,492 (10.6%)
Recurrent ED visit		
Any	9403 (67.1%)	382,303 (64%)
Self-harm	3434 (24.5%)	
Died on the same day	131 (0.0%)	

Note. Abbreviations: CAMHS, Child and Adolescent Mental Health Services; ED, Emergency Department.

At the end of follow-up, among 611,336 individuals aged 5–18 years in the analytic cohort, 1451 deaths (0.2%) were recorded. Mortality was more frequent among young people with a history of self-harm than in the comparison group (Table 2). In the self-harm group, 114 individuals (0.8%) died during follow-up, compared with 1337 individuals (0.2%) in the control group, corresponding to mortality rates of 117.6 (95% CI 97.0–141.3) and 26.5 (25.1–27.9) per 100,000 person-years, respectively.

**Table 2:** Mortality and causes of death by self-harm status.

	Self-harm (N = 14,007)	Control (N = 597,329)	Mortality rate per 100,000 person-years (95% CI)		Adjusted hazard ratio (95% CI)
			Self-harm	Control	
Primary outcome					
Suicide	43 (0.31%)	290 (0.05%)	44.37 (32.11–59.77)	5.74 (5.10–6.45)	0–1 year: 13.42 (4.74–37.97) >1 year: 4.85 (3.41–6.91)
Secondary outcomes					
Accidental death	50 (0.36%)	308 (0.05%)	51.60 (38.30–68.02)	6.10 (5.44–6.82)	0–1 year: 13.89 (6.21–31.08) >1 year: 5.46 (3.86–7.73)
Other causes of death	21 (0.15%)	739 (0.12%)	21.67 (13.41–33.13)	14.64 (13.60–15.73)	1.09 (0.70–1.69)
All-cause mortality	114 (0.81%)	1337 (0.22%)	117.64 (97.04–141.32)	26.48 (25.08–27.94)	3.11 (2.56–3.79)

Note. Adjusted hazard ratios were estimated using Cox proportional hazards model adjusted for age and sex. Where proportional hazards assumptions were violated, time-dependent effects (0–1 year and >1 year) and/or stratification by age group (5–9, 10–14 and 15–18 years) were applied. Mortality rates are reported per 100,000 person-years with 95% confidence intervals derived from Poisson models. Cause-specific analyses censored competing causes of death.

Suicide accounted for a larger proportion of deaths among individuals with self-harm than among controls (43 of 114 [37.7%] vs 290 of 1337 [21.7%]), and accidental deaths were also more frequent in the self-harm group (50 of 114 [43.9%] vs 308 of 1337 [23.0%]). By contrast, deaths due to other causes made up a substantially greater proportion of deaths in the control group than in the self-harm group (739 of 1337 [55.3%] vs 21 of 114 [18.4%]; Fig. 2). Cause-specific mortality rates were consistently higher among individuals with self-harm than controls, particularly for suicide and accidental death (Table 2).

**Fig. 2:**
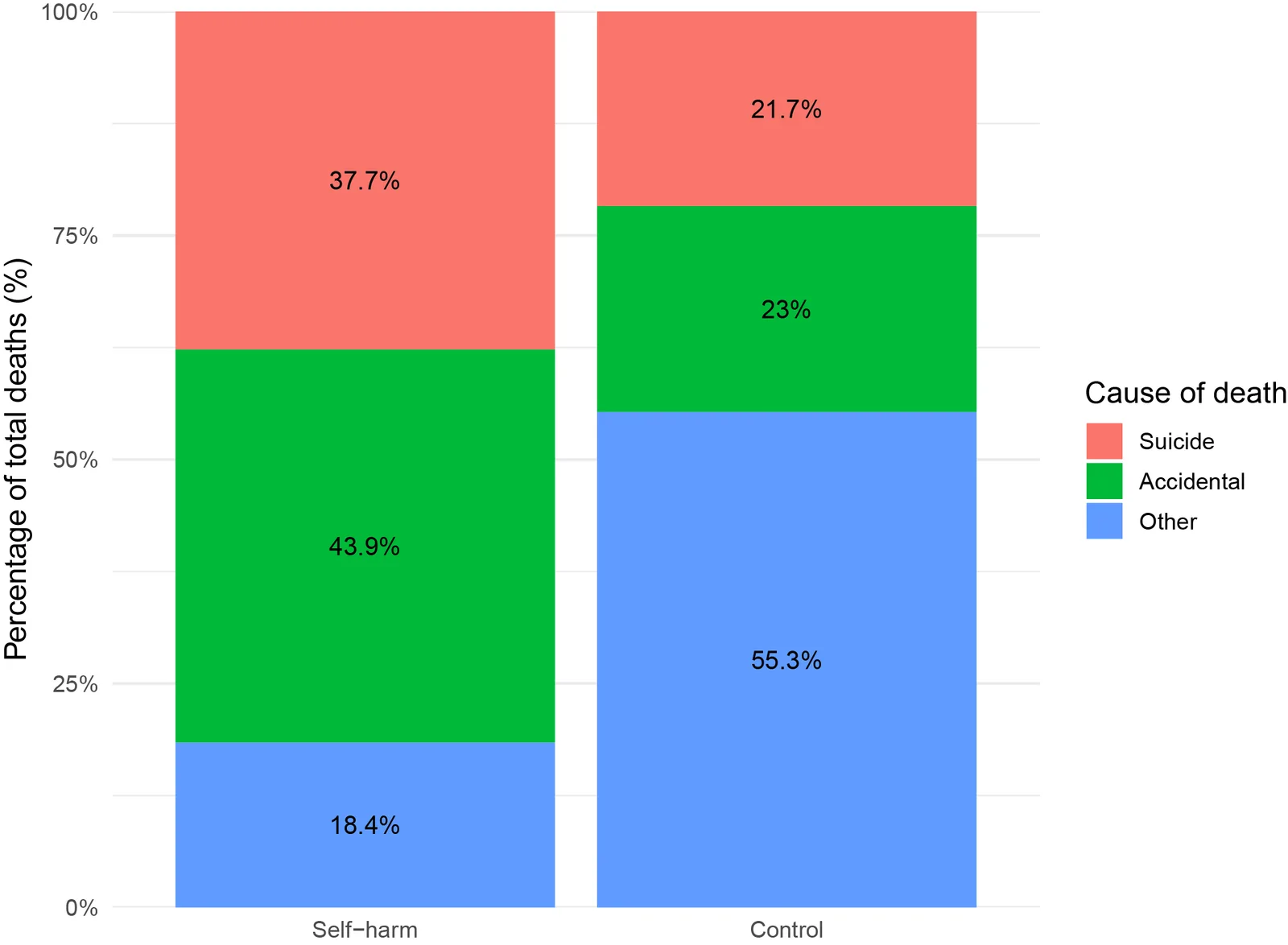
**Cause of death distribution, by self-harm status.** Note. Abbreviation: ED, Emergency Department.

In survival analyses adjusted for age and sex, the association between self-harm and suicide risk varied over time. The hazard of suicide was markedly elevated during the first year following the index ED presentation (aHR 13.42, 95% CI 4.74–37.97) and remained increased beyond one year (aHR 4.85, 95% CI 3.41–6.91). A similar time-dependent pattern was observed for accidental death, with a substantially increased hazard in the first year (aHR 13.89, 95% CI 6.21–31.08) that persisted, albeit at a lower magnitude, thereafter (aHR 5.46, 95% CI 3.86–7.73). By contrast, there was no clear association between self-harm and mortality due to other causes (aHR 1.09, 95% CI 0.70–1.69). Overall, self-harm was associated with more than a threefold increased hazard of all-cause mortality compared with the control group (aHR 3.11, 95% CI 2.56–3.79; Table 2 and Fig. 3). Findings were similar after additional adjustment for other demographic and clinical characteristics (Supplementary Table S1).

**Fig. 3:**
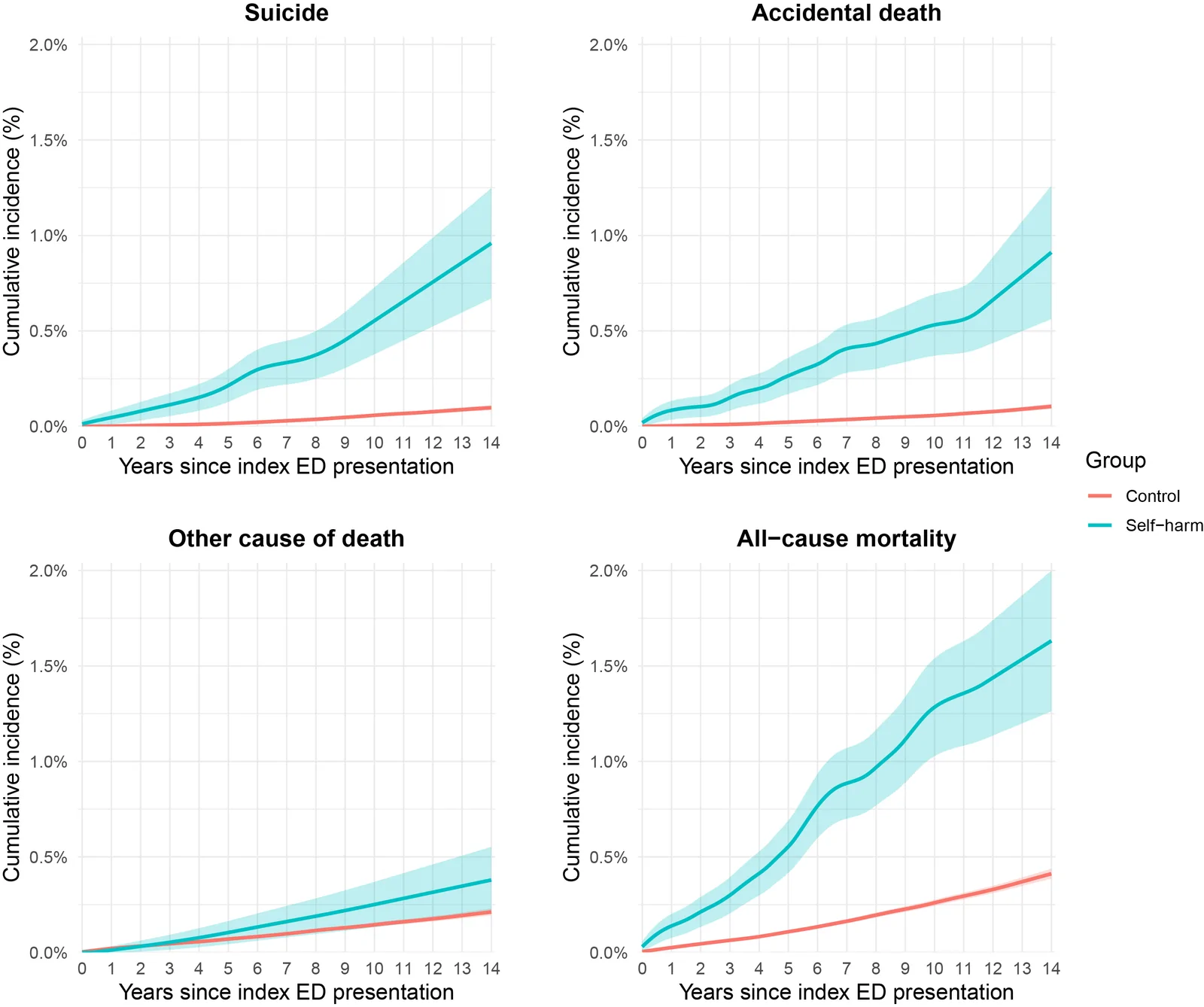
**Cumulative incidence of mortality following emergency department presentation**.

Median time to death and age at death are summarised in Table 3 and Fig. 4. Median time to death was shorter in the self-harm group than in the control group for suicide (4.9 vs 7.3 years), accidental death (4.3 vs 6.3 years), and all-cause mortality (4.5 vs 6.0 years; Table 3). Deaths were concentrated among individuals whose index ED presentation occurred in later adolescence (15–18 years). No suicide deaths were observed following a first self-harm presentation among children aged 5–9 years, although event counts in this age group were small.

**Table 3:** Median time to event (IQR) by self-harm status.

	Median time to event, years (IQR)	
	Self-harm	Control
Primary outcome		
Suicide	4.85 (2.14–5.89)	7.32 (4.77–9.77)
Secondary outcomes		
Accidental death	4.32 (1.57–6.35)	6.34 (3.92–9.63)
Other causes of death	5.20 (3.18–6.33)	5.02 (1.98–8.18)
All-cause mortality	4.49 (2.07–6.30)	5.99 (2.87–8.91)

Note. Abbreviation: IQR, Interquartile Range.

**Fig. 4:**
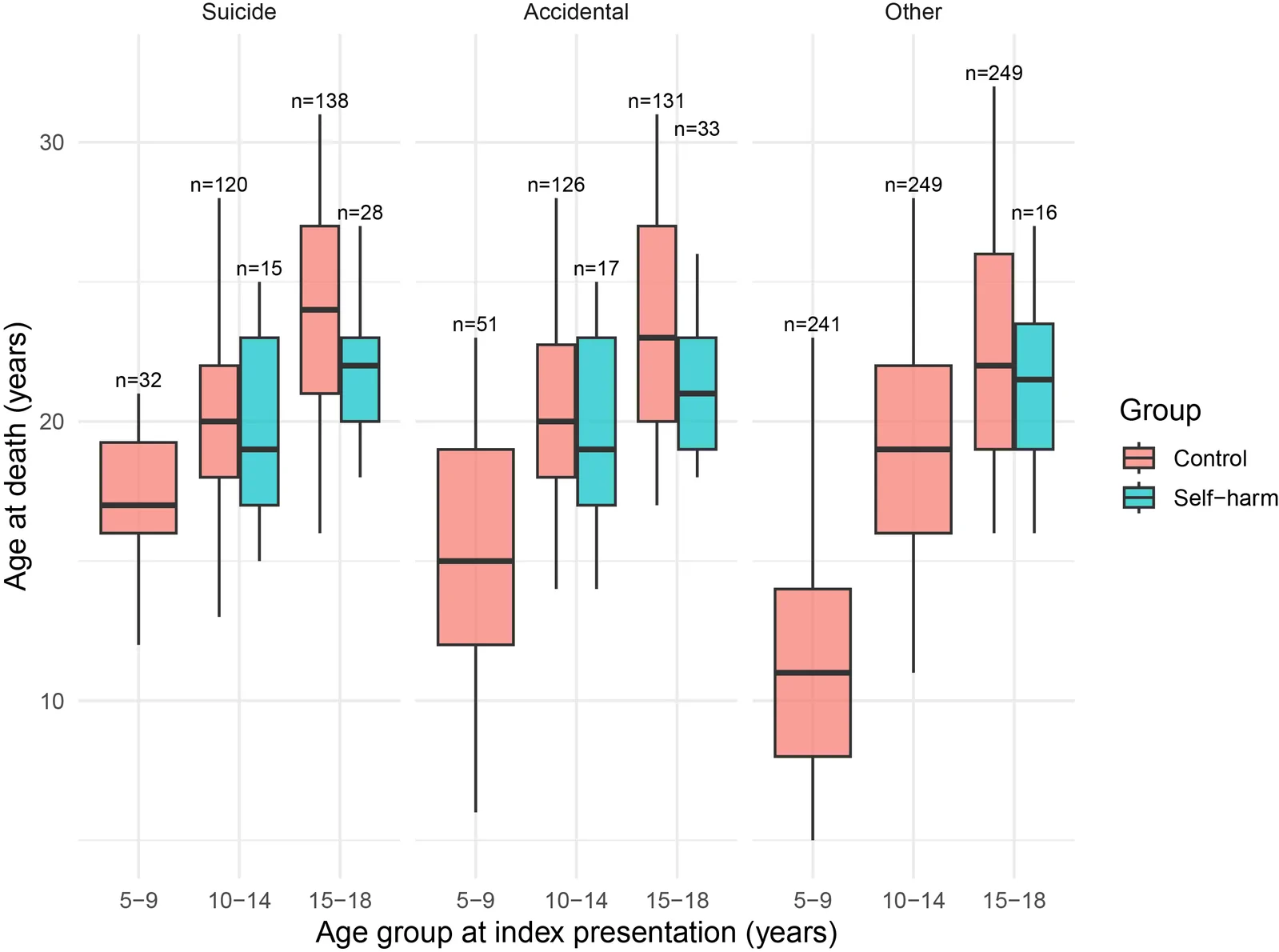
**Age at death by age group at index ED presentation**.

Within the self-harm group, several demographic and clinical characteristics were associated with subsequent mortality (Table 4). Older age at index presentation was associated with higher risks of suicide (adjusted IRR 1.27, 95% CI 1.05–1.54) and all-cause mortality (1.22, 1.09–1.36). Female sex was associated with substantially lower risks of suicide (0.39, 0.21–0.72), accidental death (0.31, 0.17–0.56), and all-cause mortality (0.39, 0.26–0.57). Recurrent self-harm was strongly associated with suicide (3.15, 1.66–5.99) and all-cause mortality (1.77, 1.17–2.68), but not with accidental or other-cause mortality. No significant associations with mortality were observed for deprivation, urban residence, recent CAMHS contact, Looked-After Child status, or hospital admission subsequent to self-harm presentation.

**Table 4:** Risk factors for mortality within the self-harm cohort.

	Adjusted IRR (95% CI)			
	Suicide	Accidental death	Other causes of death	All-cause mortality
**Age at index** (years)	1.27 (1.05–1.54)∗	1.18 (1.00–1.38)∗	1.24 (0.96–1.60)	1.22 (1.09–1.36)∗∗∗
**Sex** (female)	0.39 (0.21–0.72)∗∗	0.31 (0.17–0.56)∗∗∗	0.64 (0.27–1.55)	0.39 (0.26–0.57)∗∗∗
**Deprivation** (1 = most deprived to 5 = least deprived; per quintile increase)	1.09 (0.88–1.36)	0.92 (0.74–1.14)	0.77 (0.53–1.10)	0.96 (0.83–1.10)
**Urban residence**	1.07 (0.52–2.19)	0.71 (0.38–1.32)	0.44 (0.18–1.10)	0.75 (0.50–1.14)
**CAMHS contact** (previous 12 months)	1.07 (0.55–2.06)	1.09 (0.58–2.02)	0.54 (0.18–1.63)	0.96 (0.64–1.46)
**Looked-After child status**	1.25 (0.58–2.69)	1.39 (0.68–2.85)	2.63 (0.98–7.04)	1.51 (0.95–2.39)
**Hospital admission** (within 7 days)	0.83 (0.44–1.57)	1.12 (0.63–2.01)	1.38 (0.56–3.35)	1.04 (0.70–1.53)
**Recurrent self-harm**	3.15 (1.66–5.99)∗∗∗	1.27 (0.65–2.48)	1.02 (0.36–2.95)	1.77 (1.17–2.68)∗∗

Note. Adjusted incidence rate ratios (IRRs) were estimated using Poisson regression with log(person-time) as an offset, including all predictors simultaneously. Analyses were restricted to individuals with positive follow-up time. Significance: ∗p < 0.05, ∗∗p < 0.01, ∗∗∗p < 0.001.

Sensitivity analyses examined the impact of reclassifying deaths of undetermined intent and modelling recurrent self-harm as a time-varying exposure. Reclassifying deaths of undetermined intent as accidental attenuated estimates for suicide while strengthening associations with accidental mortality, without materially altering conclusions regarding excess mortality following self-harm (Supplementary Table S2). Modelling recurrent self-harm as a time-varying exposure showed increased suicide risk following repeat presentations, with the highest risk after recurrence (Supplementary Figure S1).

## Discussion

Children and adolescents presenting to EDs with self-harm had more than a threefold increased hazard of all-cause mortality than those presenting for other reasons. In the first year after self-harm presentation, suicide risk was more than 13 times higher and mortality from accidents around 14 times higher among young people who self-harmed compared with those presenting to ED for other reasons. Although risks were highest in the first year, both suicide and accidental mortality remained substantially elevated beyond one year, indicating a persisting vulnerability. These findings are consistent with previous studies demonstrating elevated risks of suicide and accidental death following self-harm in young people.^2^^,^^7^, ^8^, ^9^, ^10^ Our study extends this literature by using a large national cohort, including younger children, incorporating long-term follow-up, and simultaneously examining suicide, accidental mortality, and all-cause mortality within a single population-based cohort. Taken together with previous work from the UK, Ireland, and Canada, our findings provide further evidence that ED presentation for self-harm identifies a population at sustained risk of premature mortality across multiple causes of death.

The observed differences in age distribution between groups likely reflect, in part, the cohort definition, with the index date defined as the first self-harm presentation for exposed individuals and the first ED presentation for the comparison group. However, the observed associations remained robust after adjustment for age and sex. Residual confounding due to differences in baseline characteristics and unmeasured psychosocial factors cannot be excluded.

From a clinical perspective, ED presentation for self-harm identifies a group of young people at particularly high risk and therefore represents a critical window for intervention. Our findings suggest that risk assessment and post-presentation care for young people who self-harm should extend beyond suicide prevention alone to address broader risks of premature mortality, including accidental death. This is particularly relevant in the first year after presentation, when excess mortality risk was highest, and among individuals with recurrent self-harm presentations. Recent evidence indicates that evidence-based therapeutic interventions following self-harm in adolescents reduce repeat self-harm and suicide attempts, although a reduction in mortality has not yet been demonstrated, likely reflecting the rarity of this outcome in intervention trials.^31^ Complementing this evidence, recommendations for the management of paediatric mental and behavioural health emergencies emphasise the importance of coordinated emergency care, structured discharge planning, and timely linkage to outpatient services, underscoring the need for robust follow–up pathways after ED presentation.^32^ Nevertheless, most suicide deaths in our cohort occurred among young people without a prior ED presentation for self-harm, highlighting that although ED-based interventions represent a critical component of prevention, broader population- and community-based strategies remain essential to identify and support young people who never present to emergency services.

We did not find clear associations between certain deprivation factors (including area-level and Looked-After Child status) and suicide. Although these findings suggest that these factors may have limited prognostic value among young people already presenting to EDs with self-harm, they should not be interpreted as diminishing the importance of broader public health approaches. Rather, our findings support the need for multilevel suicide prevention strategies that combine population-level interventions with individualised assessment and follow-up of young people presenting with self-harm.^33^ Future research should evaluate whether prognostic models can provide an evidence-based framework to support clinical decision-making following self-harm,^34^ including linkage to services, follow-up intensity, and treatment planning. Risk stratification approaches will require prospective validation before implementation in routine clinical practice, particularly given current pressures on CAMHS services.

The observed association between self-harm and accidental death may also reflect shared underlying vulnerabilities rather than a direct effect of self-harm itself. Self-harm in young people frequently co-occurs with impulsivity, substance use, risk-taking behaviours, and psychosocial adversity, all of which may contribute to both self-harm presentations and subsequent accidental mortality.^2^^,^^6^, ^7^, ^8^ Although the mechanisms underlying this association cannot be determined from the present study, these factors represent plausible explanations for the observed association. These pathways may persist beyond the immediate post-presentation period and help explain the sustained elevation in accidental mortality observed in this study. Although some misclassification of intent is possible, whereby deaths recorded as accidental might represent unrecognised suicides, the magnitude and persistence of the association suggest that accidental mortality following self-harm is a clinically meaningful outcome in its own right.

No deaths were observed among children whose first ED presentation for self-harm occurred at ages 5–9 years, although the numbers in this age group were small. Inclusion of younger children was informed by meta-analytic evidence indicating that suicidal ideation and self-harm behaviours in childhood are more prevalent than previously recognised.^11^^,^^12^ Evidence in this age range remains limited, underscoring the need for further research.

Several features strengthen the validity of these findings, including the large, population-based cohort, long follow-up period, inclusion of younger children, and near-complete national coverage of ED presentations in Wales. To our knowledge, this is one of the largest studies to date linking ED presentations for self-harm in children and adolescents to subsequent cause-specific mortality. The use of linked, routinely collected data enabled comprehensive ascertainment of deaths and extended follow-up beyond adolescence into early adulthood. Sensitivity analyses examining alternative outcome classifications and recurrent self-harm as a time-varying exposure yielded findings consistent with the corresponding primary analyses. The latter demonstrated that recurrent self-harm presentations were associated with a further increase in mortality risk beyond the initial presentation, supporting the robustness of the observed associations and reinforcing the importance of recurrent presentations as markers of particularly high risk.

Our study has some limitations. First, ICD-10 diagnostic codes were not available for ED presentations; identification of self-harm therefore relied on a previously validated classification approach based on ED attendance and diagnostic groupings.^22^^,^^23^ Thus, some self-harm presentations—particularly those not conforming to conventional clinical coding patterns or presenting without overt injury or poisoning—may have been missed,^35^ and stratification by method or intent was not possible despite its clinical relevance.^6^^,^^8^^,^^36^, ^37^, ^38^ These limitations are consistent with those reported in other registry-based studies using routinely collected emergency care data. More broadly, surveillance of self-harm in emergency settings remains constrained by the absence of comprehensive national recording systems and persistent challenges in accurate ascertainment within routinely collected health data.^38^ Second, although ICD-10 codes were available for causes of death, low event counts in certain subgroups—notably among younger children and for cause-specific outcomes—limited further stratification beyond suicide, accidental death, and other causes. This reduced the precision of some estimates and necessitated the aggregation of categories. Sex stratified analyses were also not feasible due to these low event counts. Similarly, the low number of mortality events precluded more flexible modelling of changes in incidence rates over follow-up; therefore, IRRs from the Poisson models should be interpreted as average rates across the follow-up period. The association between ED presentation for self-harm and mortality may vary between boys and girls, and sex-specific associations are an important area for future research. Third, this study captured only self-harm episodes presenting to EDs and therefore did not include episodes managed in primary care, community, or mental health settings. This reflects the focus of our study on a clinically important high-risk group (ED presenters) rather than aiming to explore all population-level self-harm. Accordingly, our findings should be interpreted as applying to the subgroup of children and adolescents whose self-harm resulted in emergency department presentation, rather than to all young people who engage in self-harm, many of whom never present to emergency services. Prior registry-based research in Wales has shown divergent temporal trends in self-harm presentations across clinical services, highlighting that ED data represent only a subset of self-harm behaviour in the community.^22^ Fourth, although survival models were adjusted for age and sex, residual confounding cannot be excluded, particularly given differences in age at index presentation between exposure groups arising from cohort definition. Information on important psychosocial factors, such as family adversity, substance use, or exposure to violence, was not consistently available in routine data and could not be accounted for. In addition, changes in clinical documentation and coding practices across hospitals and over time may have influenced ascertainment, although use of a standardised national coding framework is likely to have reduced such variation. Finally, this study was conducted within the Welsh healthcare system, and findings may not be directly generalisable to settings with different patterns of emergency care use, self-harm recording practices, or post-presentation pathways.

There is a clear need for comparable population-based studies in LMICs, where the burden of self-harm and suicide is substantial, but routine surveillance systems are often limited, posing important challenges for research and prevention efforts.^14^^,^^37^ Further research is also needed to clarify developmental pathways linking suicidal ideation, suicidal behaviour, and subsequent risks of premature mortality across childhood and adolescence. Although identification of suicidal ideation is recognised as clinically important, it remains difficult in routine clinical settings, which may partly explain the scarcity of population-level evidence in this area. Notably, the limited registry-based evidence available suggests that ED presentations with suicidal ideation are themselves associated with markedly elevated risks of suicide, accidental death, and other external causes, underscoring the potential importance of early identification and follow-up in acute care settings.^39^ Further research is needed to assess the feasibility and acceptability of risk stratification and prognostic modelling in this population.^40^

Young people presenting to EDs with self-harm face substantially increased risks of suicide and accidental death compared with those presenting for other reasons, particularly in the 12 months after presentation. Prevention and follow-up care should therefore extend beyond suicide alone to address broader risks of premature mortality.

## Contributors

GMB conceived the study, conducted the statistical analyses and draughted the manuscript. KO provided input on the analytical approach. GMB and KO accessed and verified the underlying data. KO, RY, SF, and IK critically reviewed and revised the manuscript. IK co-conceived and supervised the study, oversaw the analyses, and secured funding. All authors read and approved the final manuscript.

## Data sharing statement

The data supporting the findings of this study are anonymised and held within the SAIL Databank. These data are not publicly available and may be accessed only through application to the SAIL Databank, subject to approval via its independent information governance review process (www.saildatabank.com).

## Declaration of interests

IK reports grant funding from the Academy of Medical Sciences and the UK Department for Business, Energy and Industrial Strategy. KO reports grant funding from the Royal Irish Academy, paid to the lead applicant's institution, and travel support from the Edinburgh Mental Health Network. SF reports providing expert evidence at inquests into deaths in custody and deaths of psychiatric patients in the community and serving as an expert member of the UK Independent Advisory Panel on Deaths in Custody. RY reports support from the John Fell Fund for the present work. GMB declares no competing interests.

## References

[1] National Institute for Health and Clinical Excellence (2024). Self-harm: assessment, management and preventing recurrence. https://www.nice.org.uk/guidance/ng225.

[2] Hawton K., Bale L., Brand F. (2020). Mortality in children and adolescents following presentation to hospital after non-fatal self-harm in the multicentre study of self-harm: a prospective observational cohort study. Lancet Child Adolesc Health.

[3] Hawton K., Harriss L. (2007). Deliberate self-harm in young people: characteristics and subsequent mortality in a 20-year cohort of patients presenting to hospital. J Clin Psychiatry.

[4] Gillies D., Christou M.A., Dixon A.C. (2018). Prevalence and characteristics of self-harm in adolescents: meta-analyses of community-based studies 1990–2015. J Am Acad Child Adolesc Psychiatry.

[5] Moran P., Chandler A., Dudgeon P. (2024). The Lancet Commission on self-harm. Lancet.

[6] Hawton K., Bergen H., Kapur N. (2012). Repetition of self-harm and suicide following self-harm in children and adolescents: findings from the multicentre study of Self-harm in England. J Child Psychol Psychiatry.

[7] Morgan C., Webb R.T., Carr M.J. (2017). Incidence, clinical management, and mortality risk following Self harm among children and adolescents: cohort study in primary care. BMJ.

[8] Finkelstein Y., Macdonald E.M., Hollands S. (2015). Long-term outcomes following self-poisoning in adolescents: a population-based cohort study. Lancet Psychiatry.

[9] Ross E., O'Reilly D., O'Hagan D., Maguire A. (2023). Mortality risk following self-harm in young people: a population cohort study using the Northern Ireland Registry of Self-Harm. J Child Psychol Psychiatry.

[10] Olfson M., Wall M., Wang S. (2018). Suicide after deliberate self-harm in adolescents and young adults. Pediatrics.

[11] Geoffroy M.C., Bouchard S., Per M. (2022). Prevalence of suicidal ideation and self-harm behaviours in children aged 12 years and younger: a systematic review and meta-analysis. Lancet Psychiatry.

[12] Liu R.T., Walsh R.F.L., Sheehan A.E., Cheek S.M., Sanzari C.M. (2022). Prevalence and correlates of suicide and nonsuicidal self-injury in children: a systematic review and meta-analysis. JAMA Psychiatry.

[13] Moran P., Coffey C., Romaniuk H. (2012). The natural history of self-harm from adolescence to young adulthood: a population-based cohort study. Lancet.

[14] Hawton K., Fagg J., Simkin S., Bale E., Bond A. (2000). Deliberate self-harm in adolescents in Oxford, 1985–1995. J Adolesc.

[15] Hawton K., Hall S., Simkin S. (2003). Deliberate self-harm in adolescents: a study of characteristics and trends in Oxford, 1990–2000. J Child Psychol Psychiatry.

[16] Office for National Statistics (2025). Death registration summary statistics, England and Wales: 2024. https://www.ons.gov.uk/peoplepopulationandcommunity/birthsdeathsandmarriages/deaths/bulletins/deathregistrationsummarystatisticsenglandandwales/2024.

[17] Jones K.H., Ford D.V., Jones C. (2014). A case study of the Secure Anonymous Information Linkage (SAIL) gateway: a privacy-protecting remote access system for health-related research and evaluation. J Biomed Inform.

[18] Ford D.V., Jones K.H., Verplancke J.-P. (2009). The SAIL Databank: building a national architecture for e-health research and evaluation. BMC Health Serv Res.

[19] Lyons R.A., Jones K.H., John G. (2009). The SAIL databank: linking multiple health and social care datasets. BMC Med Inform Decis Mak.

[20] Rodgers S.E., Demmler J.C., Dsilva R., Lyons R.A. (2012). Protecting health data privacy while using residence-based environment and demographic data. Health Place.

[21] Rodgers S.E., Lyons R.A., Dsilva R. (2009). Residential Anonymous Linking Fields (RALFs): a novel information infrastructure to study the interaction between the environment and individuals' health. J Public Health.

[22] Marchant A., Turner S., Balbuena L. (2020). Self-harm presentation across healthcare settings by sex in young people: an e-cohort study using routinely collected linked healthcare data in Wales, UK. Arch Dis Child.

[23] Clements C., Turnbull P., Hawton K. (2016). Rates of self-harm presenting to general hospitals: a comparison of data from the Multicentre Study of Self-Harm in England and Hospital Episode Statistics. BMJ Open.

[24] Gunnell D., Bennewith O., Simkin S. (2013). Time trends in coroners' use of different verdicts for possible suicides and their impact on officially reported incidence of suicide in England: 1990–2005. Psychol Med.

[25] O'Hare K., Chitsabesan P., Ford T.J. (2025). Trends in annual and lifetime prevalence of child and adolescent mental health service use in the UK between 1991 and 2023: welsh healthcare register linkage study. Br J Psychiatry.

[26] Mathur R., Bhaskaran K., Chaturvedi N. (2014). Completeness and usability of ethnicity data in UK-based primary care and hospital databases. J Public Health.

[27] Schoenfeld D. (1982). Partial residuals for the proportional hazards regression model. Biometrika.

[28] Snowdon J., Choi N.G. (2020). Undercounting of suicides: where suicide data lie hidden. Glob Public Health.

[29] Björkenstam C., Johansson L.-A., Nordström P. (2014). Suicide or undetermined intent? A register-based study of signs of misclassification. Popul Health Metr.

[30] von Elm E., Altman D.G., Egger M. (2007). The Strengthening the Reporting of Observational Studies in Epidemiology (STROBE) statement: guidelines for reporting observational studies. PLoS Med.

[31] Mughal F., Young P., Stahl D., Asarnow J.R., Ougrin D. (2025). Mortality in adolescents after therapeutic intervention for self-harm: a systematic review and meta-analysis. JCPP Adv.

[32] Hoffmann J.A., Foster A.A., Krass P. (2026). A research agenda for acute pediatric mental and behavioral health emergencies. Ann Emerg Med.

[33] Favril L., Yu R., Uyar A., Sharpe M., Fazel S. (2022). Risk factors for suicide in adults: systematic review and meta-analysis of psychological autopsy studies. Evid Based Ment Health.

[34] Fazel S., Vazquez-Montes M.D.L.A., Lagerberg T. (2024). Risk of repeat self-harm among individuals presenting to healthcare services: development and validation of a clinical risk assessment model (OxSET). BMJ Ment Health.

[35] Knipe D., Padmanathan P., Newton-Howes G., Chan L.F., Kapur N. (2022). Suicide and self-harm. Lancet.

[36] Beckman K., Mittendorfer-Rutz E., Waern M., Larsson H., Runeson B., Dahlin M. (2018). Method of self-harm in adolescents and young adults and risk of subsequent suicide. J Child Psychol Psychiatry.

[37] Grover C., Huber J., Brewer M., Basu A., Large M. (2023). Meta-analysis of clinical risk factors for suicide among people presenting to emergency departments and general hospitals with suicidal thoughts and behaviours. Acta Psychiatr Scand.

[38] Griffin E., Corcoran P., Arensman E., Kavalidou K., Perry I.J., McMahon E.M. (2023). Suicide risk following hospital attendance with self-harm: a national cohort study in Ireland. Nat Ment Health.

[39] Ross E., Murphy S., O'Hagan D., Maguire A., O'Reilly D. (2023). Emergency department presentations with suicide and self-harm ideation: a missed opportunity for intervention?. Epidemiol Psychiatr Sci.

[40] Oliver D., Beaudry G.M., Coutts F., Fazel S. (2026). Emerging opportunities and challenges for clinical prediction models in psychiatry: commentary, Oliver et al. Br J Psychiatry.

