# Supplementary material for: Risk of mortality after emergency department presentation for self-harm among children and adolescents in Wales: a population-based cohort study
Source: eClinicalMedicine. 2026 Sep 28;100:104230. doi: 10.1016/j.eclinm.2026.104230 (PMC13651489; doi:10.1016/j.eclinm.2026.104230)
Supplement: Supplementary file 1 [file mmc3.pdf]

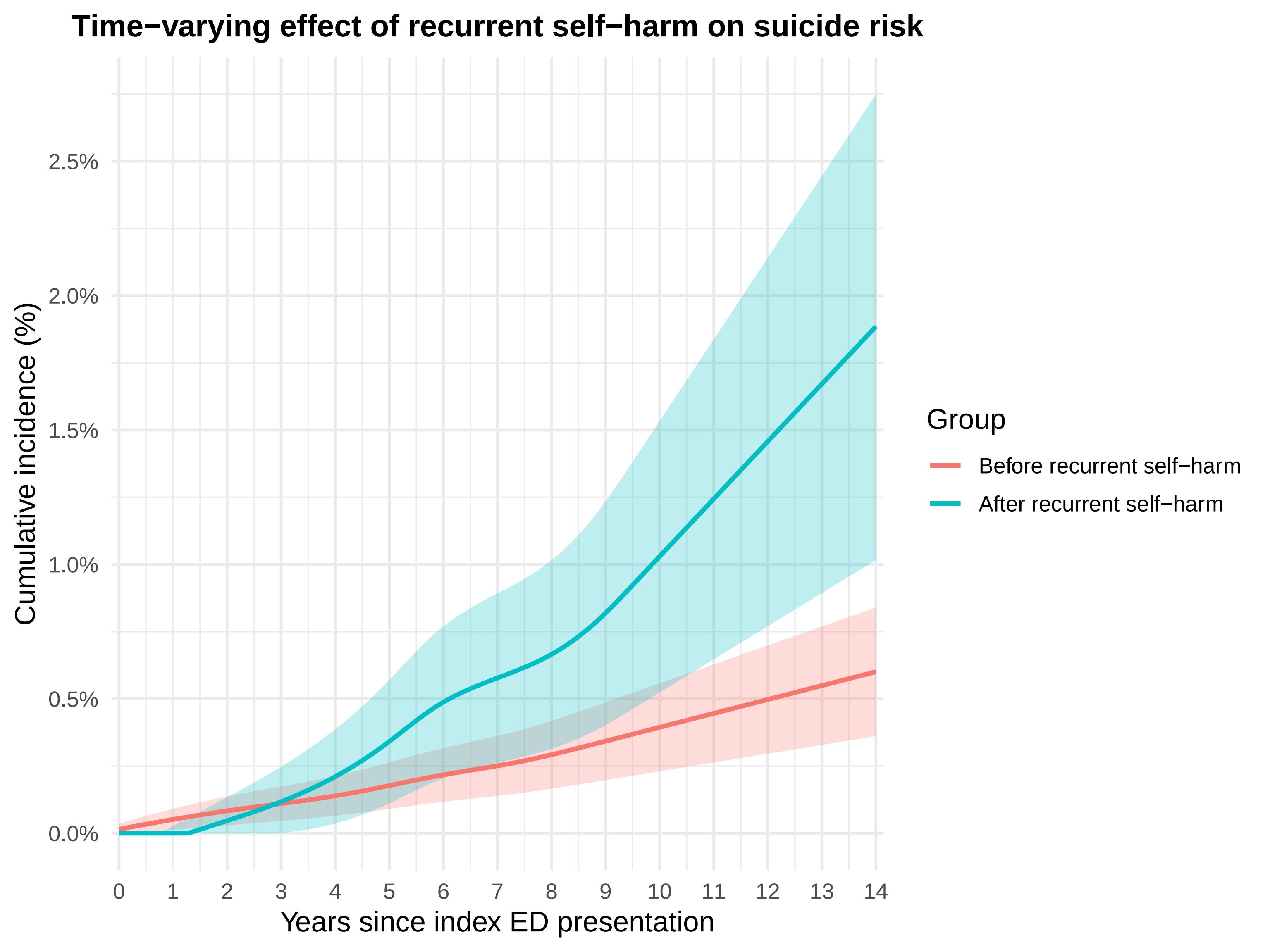
Time−varying effect of recurrent self−harm on suicide risk
Cumulative incidence (%)
0.0%
0.5%
1.0%
1.5%
2.0%
2.5%
0
1
2
3
4
5
6
7
8
9
10
11
12
13
14
Years since index ED presentation
Group
Before recurrent self−harm
After recurrent self−harm
