# Supplementary material for: Risk of mortality after emergency department presentation for self-harm among children and adolescents in Wales: a population-based cohort study
Source: eClinicalMedicine. 2026 Sep 28;100:104230. doi: 10.1016/j.eclinm.2026.104230 (PMC13651489; doi:10.1016/j.eclinm.2026.104230)
Supplement: Supplementary file 2 [file mmc1.docx]

**Supplementary Table 1**. Mortality and causes of death of study participants

|  | Adjusted age+sex  hazard ratio (95% CI) | Adjusted all  hazard ratio (95% CI) |
| --- | --- | --- |
|  |  |  |
| **Primary outcome** |  |  |
| Suicide | 0-1 year: 13.42 (4.74-37.97)  1 year: 4.85 (3.41-6.91) | 0-1 year: 9.71 (2.84-33.16)  1 year: 3.21 (2.15-4.79) |
| **Secondary outcomes** |  |  |
| Accidental death | 0-1 year: 13.89 (6.21-31.08)  1 year: 5.46 (3.86-7.73) | 0-1 year: 9.12 (3.71-22.43)  1 year: 4.33 (2.93-6.40) |
| Other causes of death | 1.09 (0.70-1.69) | 0.42 (0.26-0.66) |
| All-cause mortality | 3.11 (2.56-3.79) | 1.60 (1.29-1.99) |

Note. Adjusted all models include age, sex, deprivation, urbanicity, CAMHS contact, looked after child status and hospital admission
