# Supplementary material for: Risk of mortality after emergency department presentation for self-harm among children and adolescents in Wales: a population-based cohort study
Source: eClinicalMedicine. 2026 Sep 28;100:104230. doi: 10.1016/j.eclinm.2026.104230 (PMC13651489; doi:10.1016/j.eclinm.2026.104230)
Supplement: Supplementary file 3 [file mmc2.docx]

**Supplementary Table 2**. Sensitivity analysis after reclassification of deaths of undetermined intent as accidental deaths

|  |  |  | **Mortality rate per 100,000 person-years (95% CI)** | |  |
| --- | --- | --- | --- | --- | --- |
|  | **Self-harm (N=14007)** | **Control (N=597329)** | **Self-harm** | **Control** | **Adjusted hazard ratio (95% CI)** |
| **Primary outcome** |  |  |  |  |  |
| Suicide | 32 (0.23%) | 237 (0.04%) | 33.02 (22.59-46.62) | 4.69 (4.12-5.33) | 4.98 (3.40-7.30) |
| **Secondary outcomes** |  |  |  |  |  |
| Accidental death | 61 (0.44%) | 361 (0.06%) | 62.95 (48.15-80.86) | 7.15 (6.43-7.93) | 0-1 year: 16.22 (7.78-33.81)  >1 year: 5.40 (3.93-7.41) |
| **Note**. Deaths of undetermined intent (ICD-10 Y10-Y34) were reclassified as accidental deaths. Adjusted hazard ratios were estimated using Cox proportional hazards model adjusted for age and sex. Where proportional hazards assumptions were violated, time-dependent effects (0-1 year and >1 year) and/or stratification by age group (5-9, 10-14 and 15-18 years) were applied. Mortality rates are reported per 100,000 person-years with 95% confidence intervals derived from Poisson models. Cause-specific analyses censored competing causes of death. | | | | | |
